# Supplementary material for: Influence of Alternate Hot and Cold Thermal Stimulation in Cortical Excitability in Healthy Adults: An fMRI Study
Source: J Clin Med. 2019 Dec 19;9(1):18. doi: 10.3390/jcm9010018 (PMC7019540; doi:10.3390/jcm9010018)
Supplement: Supplementary file 1 [file jcm-09-00018-s001.pdf]

Supplemental Material

Brain activation map: The activation maps of *pre-alt*TS and *post-alt*TS for four conditions are shown in Figure. Activation responses varied by noxious TS and hand lateralisation.

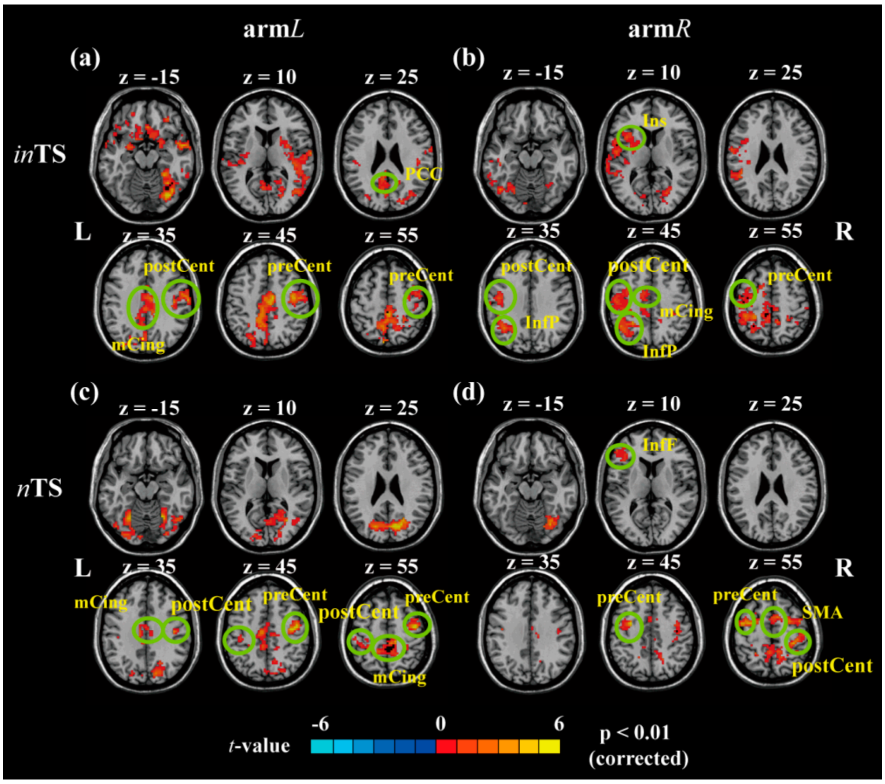

Figure S1. The activation maps of subjects for each particular condition (2 factors: arm and TS) before and after thermal stimulation. In the (**a**) arm*L*-*in*TS condition (upper-left map), the activated areas included the ventral part of frontal lobe, posterior cingulate cortex, left precentral cortex, left postcentral cortex and middle cingulate gyrus. In the (**b**) arm*R*-*in*TS condition, the aforementioned motor-related areas, insula and left inferior parietal lobe were activated. In the (**c**) arm*L*-*n*TS and (**d**) arm*R*-*n*TS conditions, a bilateral activation in the precentral cortex, postcentral cortex (S1), supplementary motor cortex and superior parietal lobe (S2) was shown. The statistical significance threshold was set at $p < 0.01$ for multiple comparisons.
